# Control of Silver Micro-Flakes Sintering and Connection Properties of Epoxy-Based Conductive Adhesives by the Effectiveness of Binder Chemistry

**DOI:** 10.3390/ma18020217

**Published:** 2025-01-07

**Authors:** Takanori Fukushima, Masahiro Inoue

**Affiliations:** Graduate School of Science and Technology, Gunma University, Ota 373-0057, Japan; t242b007@gunma-u.ac.jp

**Keywords:** electrically conductive adhesives, electrical conductivity, low-temperature sintering, binder chemistry, adhesive interface, interfacial electrical resistivity, bonding strength

## Abstract

Bonding materials with high thermal and electrical conductivity and reliable resistance to thermal stress are required. The authors have been conducting fundamental research on sintering-type bonding, in which metal micro-fillers are low-temperature sintered in the resin-bonded type electrically conductive adhesives (ECAs), as a new bonding technology, with the aim of easing thermal stress through the resin binder. This study investigated the influence of the kind of additive diluent in epoxy-based ECAs containing silver (Ag) micro-flakes on the microstructure development in the adhesives and the connection properties to metal electrodes. As a result, the sintering of Ag micro-flakes was observed to proceed in the adhesive once cured at 150 °C and by post-annealing at 250 °C. Furthermore, the sintering behavior varied greatly depending on the kind and composition of the binder additive diluent, with corresponding changes in electrical conductivity and connection characteristics with metal electrodes. Additionally, electrode surface conditions affected the connection performance. These findings are valuable for designing sintering-type bonding using resin-bonded ECAs, optimizing interfacial interactions between binder chemicals and metals.

## 1. Introduction

To address the increase in heat generation and heat density that occurs with the achievement of higher performance and miniaturization of electronic devices, there is a need for bonding materials with heat resistance and excellent electrical and thermal conductivity. To meet these performance requirements, sintered bonding materials are being considered in which metal particles are sintered [1]. Conductive pastes that contain metal nanoparticles and do not contain resin binders are widely considered to be sintered bonding materials. However, these bonding materials present challenges, such as improving bonding reliability by easing thermal stresses in the connection area due to temperature changes [2,3,4].

In expectation of a solution to these problems, this work conducts a fundamental study for sintering-type bonding in which metal micro-particles are sintered at low temperatures in the resin-bonded type electrically conductive adhesives (ECAs). Conventional ECAs are composites composed of resin binders containing conductive fillers, which form conducting contacts at the interface through an insulative barrier, making conduction paths of electrons [5]. The tunneling mechanism is believed to occur through the barrier to obtain electrical conductivity [5]. The direct bonding between fillers was proposed using metallurgical approaches to reduce the interfacial electrical resistance between fillers for enhancing the electrical conductivity of ECAs. For example, melting and solidifying metallic particles with low melting temperatures mixed as secondary fillers can be induced during curing ECAs to form direct bonding between fillers [6]. Recently, exploratory research applying Ga-based liquid metals was also reported for ECAs [7]. 

For the sintering phenomenon of metal filler in resin binders, research has been conducted on using metal nanoparticles to promote sintering [8], and adding resin to sintering-type bonding materials using nanoparticles has also been studied [9,10]. The sintering phenomenon is also reported in ECAs containing some Ag-plated resin fillers [11]. In addition, methods such as adding organometallic compounds for low-temperature sintering of metal micro-fillers are being investigated [12]. Although organic adsorbates are known to accelerate the diffusion of metal atoms at low temperatures [13], inducing metal micro-particle sintering in resin-bonded type ECAs has rarely been investigated for sintering-type bonding due to a lack of information about interphase [14,15] formed around fillers.

The key to improving connection properties is bonding material designs, which consider the adhesive interfacial properties with the metal electrode. As the analytical approaches, several non-destructive inspection methods for imaging interfacial defects, such as X-ray, scanning acoustic microscopy, and lock-in thermography, have been successfully established for quality control of ECA joints [2,16]. A destructive method using focused ion beam-scanning electron microscopy (FIB-SEM) can also provide 3D image information of filler dispersion near the interface between ECA and metal electrodes to apply various modeling works. A study has been conducted to analyze the interfacial electrical resistance between conductive adhesive and metal electrodes using a model based on 3D observation of filler dispersion near the adhesive interface [17]. In addition, a previous work of resin-bonded type ECAs suggested chemical components such as binder components and filler surface treatment agents [18] affect the interfacial electrical resistance between the ECAs and metal electrodes as well as between fillers as an interface chemical factor, from the viewpoint of experimental approaches. Despite progress in the inspection methods, the effects of the binder composition and the condition of the metal electrode surface on the interfacial electrical resistance and the interfacial microstructure near the electrode surface have been still unclear. Combining the analytical and the experimental approaches will contribute to achieving advanced interconnection technology using ECAs.

This work prepared samples of epoxy-based ECAs with silver (Ag) micro-flakes as filler by adding two types of diluents and evaluated the effects of binder chemistry and metal electrode surface properties on the interfacial microstructure and electrical and mechanical connection properties of the adhesive/metal electrodes interface. The sintering behavior of Ag micro-fillers in the resin binder was also investigated, including the interface with the copper (Cu) electrode, to determine the effect of binder formulation composition on microstructural changes and electrical properties when conductive adhesive samples were annealed at high temperature after curing.

As a result, the binder chemistry can control not only the sintering behavior of the Ag micro-filler but also the interface structure with the Cu electrode and the interface electrical resistance change. The result of this research may lead to a new development in sintering-type bonding technology, which is reported in detail below.

## 2. Materials and Methods

### 2.1. Preparation of ECA Samples

The conductive adhesive in this study consisted of 85 wt% Ag micro-flakes and 15 wt% resin binder. 4,4′-Methylenebis(N,N-diglycidylaniline) (Sigma-Aldrich Co. LCC, St. Louis, MO, USA) was used as the primary component of the resin binder. As a hardener, a curing catalyst (2E4MZ-CN, SHIKOKU KASEI Holdings Corp., Marugame, Japan) was added at 5 wt% to the binder. To these mixtures, 10.5 wt% of 2-(2-butoxyethoxy)ethanol (BC; FUJIFILM Wako Pure Chemical Corp., Osaka, Japan) was added as a non-reactive diluent to the binder, or 2,3-epoxypropyl phenyl ether (PGE; FUJIFILM Wako Pure Chemical Corp., Osaka, Japan) was added as a reactive diluent to substitute for the primary component at 20–40 wt%. Ag micro-flakes (50% Diameter: 3.5–5.5 μm, FUKUDA METAL FOIL & POWDER Co., Ltd., Kyoto, Japan) were mixed with each binder as filler to prepare ECA samples.

### 2.2. Measurement of Electrical Resistivity of ECA Samples

Prepared ECA samples were printed on a glass substrate with dimensions of 3 mm × 76 mm × 40 μm and subjected to curing and post-annealing. A total of 1 h of curing was performed in the air at 150 °C. To clarify changes after exposure to high temperatures, the cured samples were post-annealed for 1 h in the air at 190–290 °C.

The four-point probe method measured electrical resistivity after curing and post-annealing using a low-resistivity meter (MCP-T610, Nittoseiko analytech Co., Ltd., Yamato, Japan).

### 2.3. Curing Behavior Analysis of ECA Samples

To analyze the curing behavior of each conductive adhesive sample, the differential scanning calorimeter (DSC; DSC6100 or DSC6200, Hitachi High-Tech Co., Tokyo, Japan) was used to investigate the reaction heat from the curing reaction during curing and the residual exotherm of the cured sample. The temperature increase conditions were 30–200 °C at 5 °C/min for the samples in paste state and 30–300 °C at 5 °C/min for the cured samples.

### 2.4. Measurement of Electrical Resistance Changings During Post-Annealing

Changes in the electrical resistance of each conductive adhesive through post-annealing were in situ analyzed. Samples for the measurements were prepared by curing each conductive adhesive sample on a glass substrate with pre-formed four-terminal Ag electrodes. These samples were post-annealed on a heater block at 30–300 °C with a temperature increase rate of 5 °C/min. The electrical resistance change of the conductive adhesive during the process was measured in situ by the four-probe method using a digital multimeter (7461A; ADC Corp., Hiki-gun, Japan).

### 2.5. Evaluation of Electrical Connection Properties and the Changes Between Conductive Adhesive/Cu Electrode

**Figure 1:**
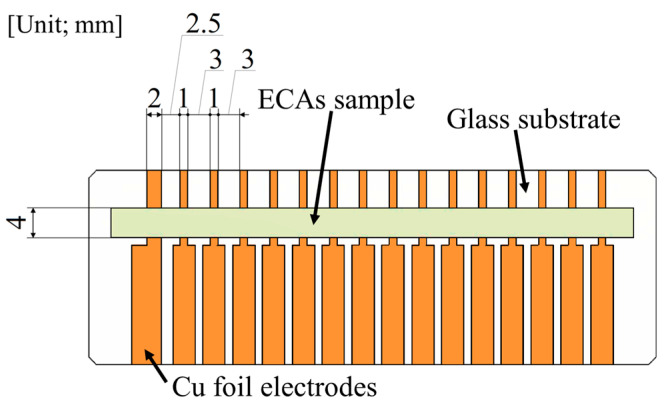
Schematic illustration of the substrate used to measure the interfacial electrical resistivity of the adhesion interface to the Cu electrode.

The electrical connection properties at the adhesion interface between each conductive adhesive and Cu electrode were evaluated by referring to ISO 16525-2 [19] for the interface electrical resistivity. The electrodes were formed using electrolytic Cu foil tape (No. 8323, Teraoka Seisakusho Co., Ltd., Tokyo, Japan) or rolled Cu foil tape (No. 8323, Teraoka Seisakusho Co., Ltd., Tokyo, Japan) on a glass substrate with dimensions as shown in Figure 1. The electrolytic Cu foil was confirmed to be treated with zinc (Zn) plating with 15–20 nm thickness using X-ray photoelectron spectroscopy (XPS) and glow discharge optical emission spectroscopy (GD-OES), as described in Appendix A. After printing the ECA samples with dimensions of 4 mm × 70 mm × 40 μm on the substrate, the samples were prepared by curing (at 150 °C for 1 h) and post-annealing (at 190–290 °C for 1 h).

After curing and post-annealing, the electrical resistance of each sample was measured at 15 points with the low-resistivity meter at different electrode distances to obtain a regression line representing the relationship between the electrical resistance and electrode distance. The electrical resistance obtained by this measurement is the sum of the electrical resistance inside the conductive paste (volume resistance, *R*_v_), the interface resistance between the paste and the electrode (*R*_i_), and the relationship between the electrical resistance of the sample (*R*) and the electrode spacing (length of adhesive, *L*) can be expressed by Equation (1):

*R* = *R*_v_ + *R*_i_ = *R*_0_ + (*ρ*/*A*) *L* (1)

where *R*_0_ is the intercept between the line and y-axis, i.e., the interfacial electrical resistance (Ω). *ρ* and *A* are the electrical resistivity and cross-sectional area of the conductive adhesive. The interfacial electrical resistivity (Ω·mm^2^) between each sample and the Cu electrode was obtained by multiplying the *R*_0_ obtained from these measurements by the area of the adhesion interface. Details of the procedure for estimating the interfacial electrical resistivity are described in Appendix B.

### 2.6. Evaluation of Mechanical Bonding Properties with Metal Electrodes

Adhesive butt-joint joints were fabricated using thin, flat Cu rivets. After each conductive adhesive was applied between two Cu rivets, the specimens were cured (at 150 °C for 1 h) and post-annealed (at 190–290 °C for 1 h) in a jig so that the thickness after curing was approximately 100 μm. After curing and post-annealing, the specimens were subjected to uniaxial tensile tests at a crosshead speed of 0.1 mm/s. Uniaxial tensile tests were performed on the cured and post-annealed specimens at a crosshead speed of 0.1 mm/s.

## 3. Results and Discussion

### 3.1. Body Characteristics Evaluation of ECA Samples

#### 3.1.1. Electrical Resistivity of Cured Samples and the Changes Through Post-Annealing

**Figure 2:**
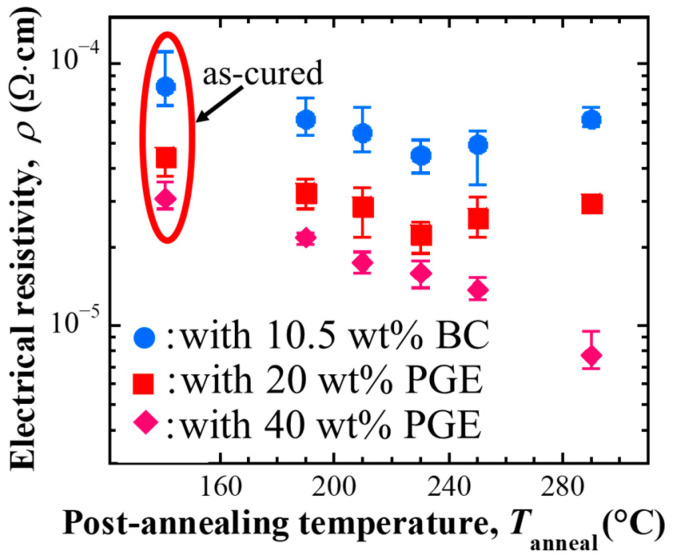
Electrical resistivity of ECA samples containing Ag flakes, comparison of before and after post-annealing [20].

The electrical resistivity of each conductive adhesive sample after curing and post-annealing was measured, as shown in Figure 2. The electrical resistivity after curing was 10^−4^–10^−5^ Ω·cm for all samples. Compared to the sample with non-reactive diluent BC, the sample with reactive diluent PGE showed decreased electrical resistivity. The electrical resistivity of the samples with PGE decreased as the amount of PGE added increased.

Regarding the electrical resistivity after post-annealing, all samples decreased compared to that after curing. When post-annealing was performed at each condition below 230 °C, the electrical resistivity tended to decrease as the post-annealing temperature increased. However, the BC-added sample and the sample with 20 wt% PGE showed a change in electrical resistivity that increased again above 250 °C. For the sample with 40 wt% PGE, the electrical resistivity decreased as a function of post-annealing temperature, and resistivity in the range of 10^−6^ Ω·cm was obtained after post-annealing at 290 °C.

#### 3.1.2. Curing Process

**Figure 3:**
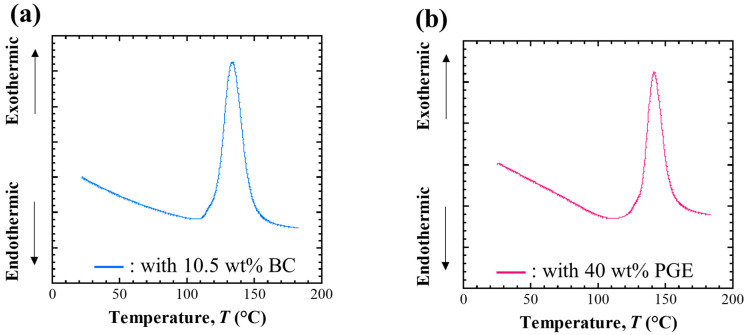
Result of DSC measurement for (**a**) ECA sample with 10.5 wt% BC and (**b**) ECA sample with 40 wt% PGE during heating from ambient temperature to 200 °C.

DSC curves from the paste state of each conductive adhesive sample were obtained, as shown in Figure 3a,b. For all samples, a clear peak related to the ring-opening of the oxirane ring by the curing reaction was observed at 130–140 °C. Furthermore, no clear reaction heat was detected when the cured samples were subjected to DSC measurements up to 300 °C. This evaluation indicates that the curing reaction proceeds completely under the curing conditions adopted in this study and that the cured samples are sufficiently cured after curing.

#### 3.1.3. Cross-Sectional Microstructure of Adhesive and Its Change Through Post-Annealing

The cross-sectional microstructure ECA samples after curing and the changes caused through the post-annealing are described here. The cross-sectional microstructure of the sample used for the interface electrical resistivity evaluation described below was observed by scanning electron microscopy (SEM; ERAX-8900M, Elionix Inc., Toyo, Japan). The microstructure of the adhesive interface and its changes are described in another section.

**Figure 4:**
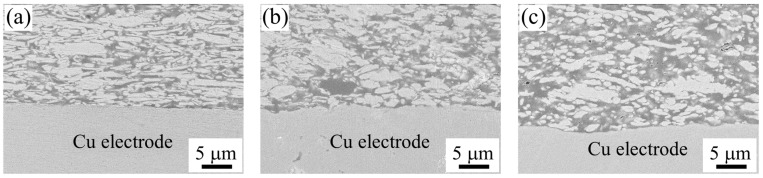
Cross-sectional SEM image of ECA samples with 10.5 wt% BC (**a**) cured at 150 °C for 1 h and post-annealed at (**b**) 250 °C, (**c**) 290 °C for 1 h on Zn plated electrolytic Cu electrode.

**Figure 5:**
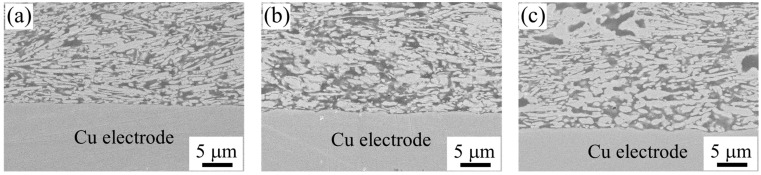
Cross-sectional SEM image of ECA samples with 40 wt% PGE (**a**) cured at 150 °C for 1 h and post-annealed at (**b**) 250 °C, (**c**) 290 °C for 1 h on Zn plated electrolytic Cu electrode.

First, SEM images of the conductive adhesive samples after curing are shown in Figure 4a and Figure 5a for the BC-added sample and the sample with 40 wt% PGE. No significant difference was observed in the microstructures of the cured samples. In both samples, Ag micro-flakes were dispersed in the resin binder in the form of flakes. In the electrical resistivity results shown earlier, a difference was observed between the BC-added and PGE-added samples. The difference is caused by a change in the interfacial electrical resistance between fillers due to binder chemistry.

Next, SEM images of the microstructure for the BC-added sample and the sample with 40 wt% PGE post-annealed at 250 and 290 °C are shown in Figure 4b,c and Figure 5b,c. First, for both samples, Ag micro-flakes were sintered using post-annealing even though no Ag nanoparticles [5,6,7] were used in the resin fully hardened by curing. The diffusion of Ag atoms can be accelerated by assisting organic molecules in forming complexes for crystal growth [13]. The sintering behavior of the fillers is assumed to occur via a similar mechanism for accelerating the diffusion of Ag atoms in the ECA samples. Although there is a lack of information about the interphase [14,15] formed around the fillers, the interfacial chemistry can affect the sintering and microstructural evolution of the ECAs.

For the BC-added sample (Figure 4b), sintering between Ag micro-flakes progressed, and coarsening of the agglomerates, which was considered Ostwald growth, was observed. When the post-annealing at 290 °C (Figure 4c), the spacing between the agglomerates expanded, and a sintered structure with scattered agglomerates was formed. As shown earlier, the increase in electrical resistivity when post-annealed at 250 °C or higher is due to the formation of such microstructure.

Sintering between Ag flakes also progressed in the PGE-added sample (Figure 5b). However, compared to the BC-added sample (Figure 4b), the Ag micro-flakes were sintered while maintaining their cross-sectional shape. The sample post-annealed at 290 °C (Figure 5c) also resulted in the formation of a continuous sintered microstructure, unlike the BC-added sample (Figure 4c), and the spacing between the agglomerates was narrower. The sintering of Ag micro-flakes was clarified to proceed in the fully once-hardened resin binder, and the sintering behavior varies depending on the binder chemistry. Since BC volatilizes during curing, almost no diluent component remains in the adhesive after curing, while PGE is a reactive diluent and remains in the adhesive except for a portion that volatilizes. Therefore, in the PGE-added sample, it is considered that the PGE remaining in the cured binder suppressed excessive microstructural evolution of Ag flakes during post-annealing. Better electrical conductivity properties can be obtained by controlling the sintering behavior of Ag micro-fillers in the resin binder through the control of binder chemistry.

#### 3.1.4. Electrical Resistance Changings During Post-Annealing

**Figure 6:**
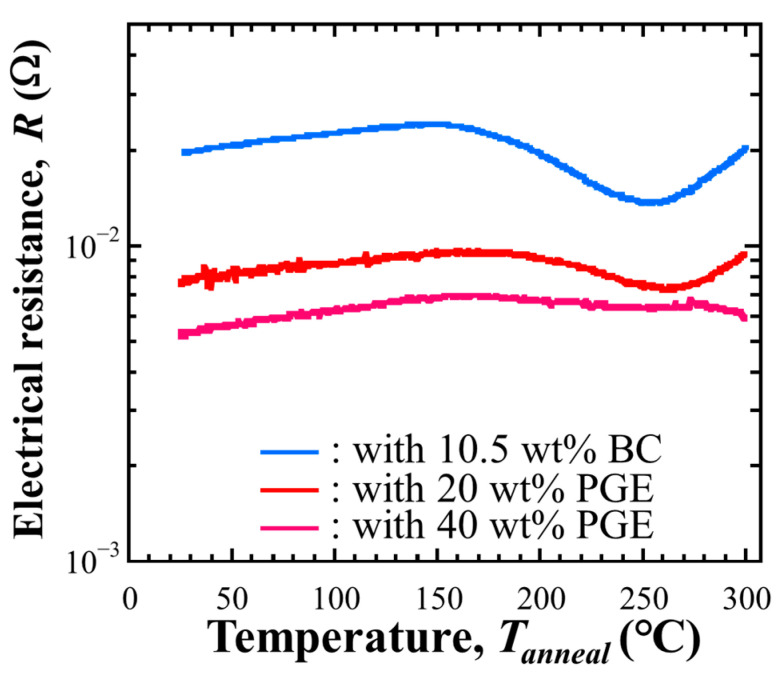
Variation in the electrical resistance of the ECA samples with non-reactive or reactive diluent during post-annealing up to 300 °C.

The changes in electrical resistance during annealing were measured for each conductive adhesive. Figure 6 shows the changes in electrical resistance when the samples were heated up to 300 °C after curing. For all composition samples, the electrical resistance increased to the curing temperature of 150 °C, and a decrease in electrical resistance was observed at temperatures above 150 °C. As the annealing temperature increased, the electrical resistance of the BC-added sample increased from about 255 °C, and that of the 20 wt% PGE-added sample increased again from about 265 °C. The electrical resistance did not increase again for the 40 wt% PGE-added main component sample and showed a gradual decrease.

### 3.2. Evaluation of Adhesion Properties at the Adhesion Interface with Cu Electrodes

#### 3.2.1. Interfacial Electrical Resistivity of the Interface Between Zn-Plated Electrolytic Cu Electrodes and Its Change

**Figure 7:**
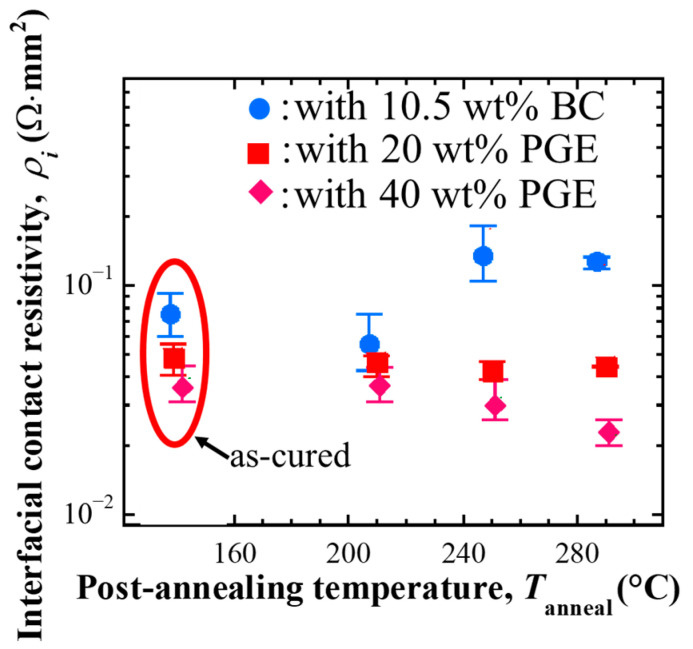
Interfacial electrical resistivity between the adhesives containing Ag flakes and Zn-plated electrolytic Cu electrode, compared before and after post-annealing [20].

Figure 7 shows the interfacial resistivity of the interface between each ECA sample and the Zn-plated electrolytic Cu electrode after curing and post-annealing.

First, the interfacial electrical resistivity after curing was lower for the PGE-added sample than for the BC-added sample, as was the resistivity inside the adhesive. In addition, the interfacial electrical resistivity decreased as the amount of PGE added increased. Added PGE acted between the fillers but also reduced the interfacial resistance with the electrodes.

Next, the interfacial electrical resistivity after post-annealing was evaluated. The sample had no significant change with 20 wt% PGE added depending on the post-annealing temperature. This change differed from the electrical resistivity inside the sample, which tended to increase with post-annealing at 250 °C or higher. For the sample with 40 wt% PGE, the interfacial electrical resistivity tended to decrease as the post-annealing temperature increased, in the same way as the electrical resistivity. On the other hand, for the BC-added samples, the interfacial electrical resistivity decreased after the post-annealing at 210 °C, but it increased significantly when the post-annealing was performed at 250 °C or higher.

#### 3.2.2. Interfacial Microstructure of the Sample with Zn-Plated Electrolytic Cu Electrodes and Its Changes

The cross-sectional microstructure of the bonding interface between the sample used for measuring interfacial electrical resistivity and the Cu electrode was observed by SEM.

Figure 4a and Figure 5a show SEM images of the BC-added sample and the sample with 40 wt% PGE after curing. There was no significant difference in the bonding interface for any of the composition samples.

Figure 4b,c show SEM images of BC-added samples after post-annealing at 250 and 290 °C, respectively. Compared to the cured sample, the surface of the Cu electrode tended to become more uneven. The BC-added samples showed that the sintering between the Ag micro-flakes in the adhesive layer progressed excessively after post-annealing. Similarly, it is thought that the excessive transfer of material via the Zn plating on the electrode surface also occurred between the Cu electrode surface and the Ag filler, causing a change in the structure that resulted in the formation of bumps on the electrode surface. The increase in interfacial electrical resistivity due to the post-annealing process described above is thought to be due to the change in the microstructure, which causes an increase in the bumps on the electrode surface and an expansion in the distance between the sintered areas due to the excessive sintering and microstructural development between the Ag flakes.

Figure 5b,c show SEM images of samples with 40 wt% PGE after post-annealing at 250 and 290 °C, respectively. Compared to the BC-added sample, almost no change in the unevenness of the Cu electrode surface was observed after curing at the interface between the conductive adhesive and the Cu electrode. In the PGE-added sample, similar to the case of Ag micro-flakes sintering as described above, it is possible that the PGE remaining in the hardened binder inhibited the mass transfer between the filler and the Zn plating on the electrode surface during the post-annealing process, preventing any changes in the unevenness. The PGE molecules are believed to segregate in the interphase formed around the Ag fillers in the ECAs [20,21,22]. Modifying the interfacial chemistry by segregating PGE molecules can control the sintering of Ag flakes.

#### 3.2.3. Interfacial Electrical Resistivity of the Interface Between Rolled Cu Electrodes and Its Change

The effect of the presence or absence of Zn plating on the Cu electrode surface on the interfacial electrical resistivity of each conductive adhesive sample to the electrode was investigated.

**Figure 8:**
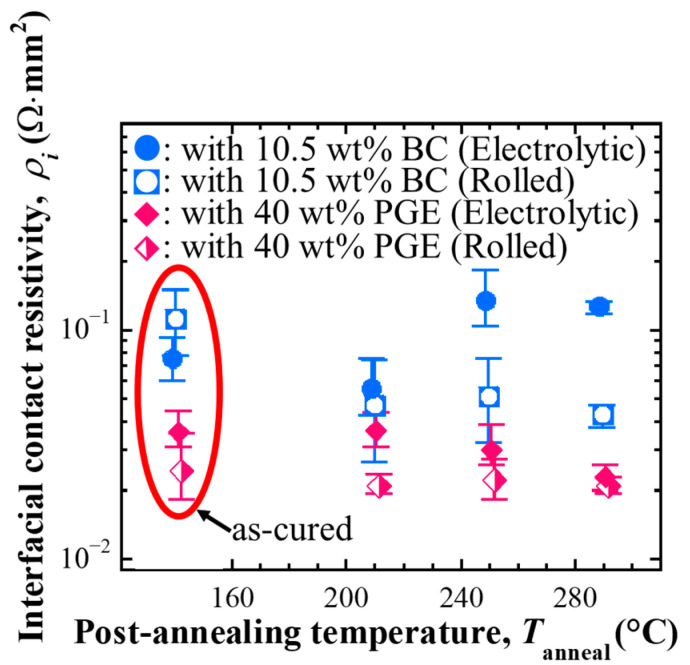
Interfacial electrical resistivity between the adhesives containing Ag flakes and Zn-plated electrolytic Cu foil electrode or rolled Cu foil electrode, comparison of before and after post-annealing [20].

Figure 8 compares the interfacial electrical resistivity of the BC-added sample and the sample with 40 wt% PGE added after curing and post-annealing at the bonding interface with the rolled Cu electrode and Zn-plated electrolytic Cu electrode. Compared to Zn-plated electrolytic Cu electrode samples, after curing, there was a slight increase in the BC-added sample and a decrease in the PGE 40 wt% added sample. The effect on the electrical connection characteristics of the bonding interface changes depending on the combination of the presence or absence of plating and the binder chemistry.

For the Zn-plated electrolytic Cu electrode samples, the BC-added samples showed a significant increase when post-annealed at 250 °C or higher, but for the rolled Cu electrode samples, there was no increase, and the value decreased depending on the post-annealing temperature. In addition, the PGE-added samples showed lower values than the Zn-plated electrolytic Cu electrode samples at all post-annealing temperatures. Still, there was no decrease in the value after curing. This indicates that the interfacial electrical resistivity was sufficiently reduced by curing.

#### 3.2.4. Interfacial Microstructure of the Sample with Rolled Cu Electrodes and Its Changes

**Figure 9:**
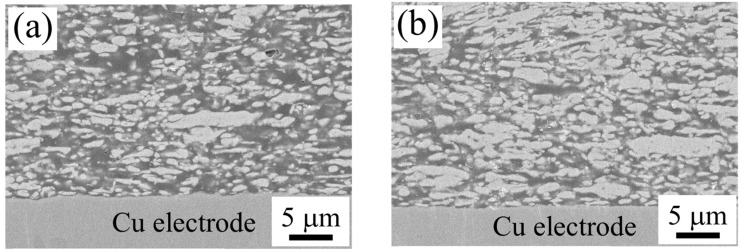
Cross-sectional SEM image of ECA samples with (**a**) 10.5 wt% BC or (**b**) 40 wt% PGE post-annealed at 290 °C for 1 h on rolled Cu electrode.

Figure 9a,b show the SEM images of the cross-sectional microstructure near the bonding interface with the rolled Cu electrode for the BC-added sample and the PGE 40 wt% main agent substitution sample, which were post-annealed at 290 °C. Comparing the interface microstructure with the Zn-plated electrolytic Cu electrode shown in Section 3.2.3, the sintering structure of the adhesive layer was found to differ depending on the binder chemistry, as with the electrolytic Cu electrode sample. However, when focusing on the interface between the electrode and the adhesive, it was confirmed that the surface of the electrode did not change much in any of the samples after curing. As described in Section 3.2.3, for the BC-added samples, the microstructure of the interface with the Zn-plated electrolytic Cu electrode changed significantly due to the post-annealing; the microstructure of the interface to the rolled Cu electrode did not change in terms of the unevenness of the electrode surface after post-annealing at 290 °C compared to after curing. From this observation, it can be thought that the increase in the unevenness of the electrode surface in the BC-added sample for the Zn-plated electrolytic Cu electrode is due to the mass transfer between the electrode and the conductive adhesive sample via the Zn plating.

Clarifying the diffusion mechanism caused by the chemical interaction between the resin and the metal is necessary in the future regarding the progress of sintering Ag micro-flakes in the hardened resin and the mass transfer between the Cu electrode and the Ag flakes via the resin binder.

#### 3.2.5. Mechanical Connection Characteristics and Changes to Metal Electrodes

**Figure 10:**
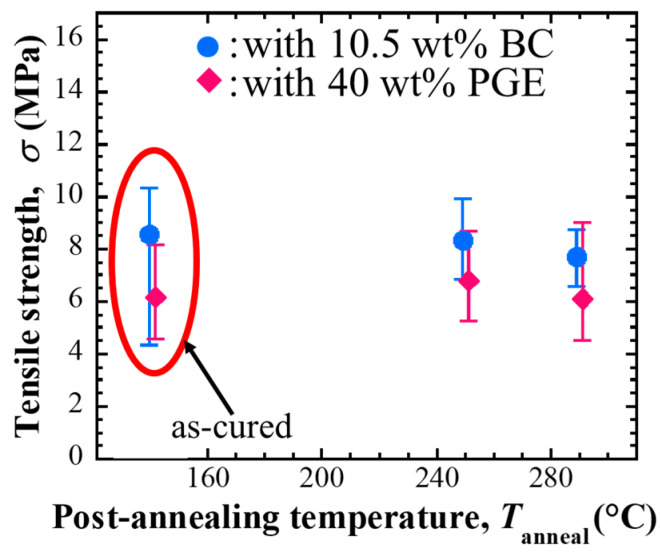
Bonding strength of Cu–Cu joints bonded with the adhesives containing Ag flakes, compared to before and after post-annealing [20].

The mechanical connection properties of each ECA sample to the Cu electrode were evaluated based on the bonding strength of adhesive butt-jointed adhesive samples. Figure 10 shows the adhesive strength of the BC-added sample and the sample with 40 wt% PGE added after curing and post-annealing. As a result, the adhesive strength after curing was 6–9 MPa, and the strength was maintained without significant decrease after post-annealing at 250 and 290 °C. Compared to the samples with non-reactive diluents, those with reactive diluents tended to have lower bonding strength. However, the main fracture mode of the samples with non-reactive diluents was interfacial failure, whereas the samples with reactive diluents showed cohesive failure, suggesting that the adhesiveness of the bonding interface is affected by the added diluents. This difference in the adhesiveness of the bonding interface may also affect the interfacial electrical resistivity mentioned above.

There is a concern that the resin-bonded conductive adhesive may deteriorate due to high-temperature post-annealing. However, the observation of the microstructure of the interface shown above did not reveal the occurrence of apparent voids or cracks in the adhesive layer or at the adhesive interface; moreover, there was no significant decrease in the adhesive strength. From these experimental facts, the deterioration of the binder, such as oxidation and decomposition, did not significantly affect the various connection characteristics under the post-annealing conditions used in this study.

## 4. Conclusions

According to this research, the sintering behavior of Ag micro-flakes in resin binders changes greatly depending on the type of additive diluent and the electrical conductivity properties change. Furthermore, it was found that the combination of the binder composition and the state of the Cu electrode surface affects the structure of the bonding interface and the connection properties. It is thought that chemical interactions occur at the interface between the organic compounds in the binder composition, the filler, and the metal electrode. By appropriately utilizing these interface chemical reactions, it is possible to promote low-temperature sintering of metal micro-fillers, control the sintering structure, and control the development of the fine structure of the bonding interface with the metal electrode. It is expected that connection properties and connection reliability will be improved. The authors intend to continue investigating the effects of each component of resin-bonded conductive adhesives to clarify the components and formulations helpful in controlling the microstructure, including the bonding interface with the metal electrode, and to establish design guidelines for the materials and bonding interface.

In some cases, the filler surface is described as chemically inert as a model for interfacial contact in conductive adhesives and at the interface with metal electrodes. However, based on the fact that the behavior of microstructure development changes depending on the binder formulation components, it can be said that the physical model of interfacial electrical contact needs to be expanded to elucidate the electrical conduction mechanism in conductive adhesives. Therefore, the study reported in this paper is essential for developing advanced conductive adhesive materials technology, and further fundamental research is required.

## Figures and Tables

**Figure A1:**
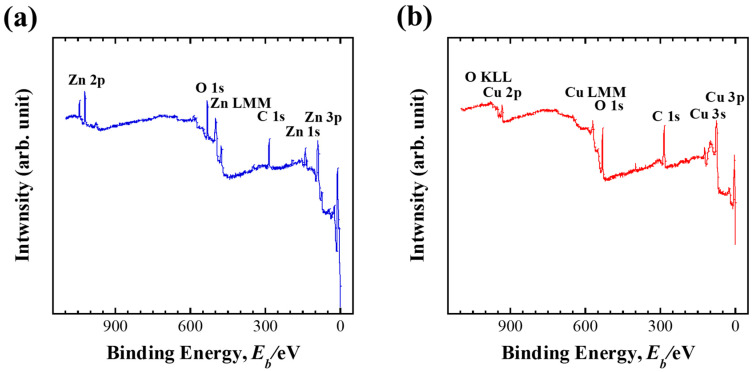
Result of XPS survey-scanning spectra of (**a**) electrolytic Cu electrode and (**b**) rolled Cu electrode.

**Figure A2:**
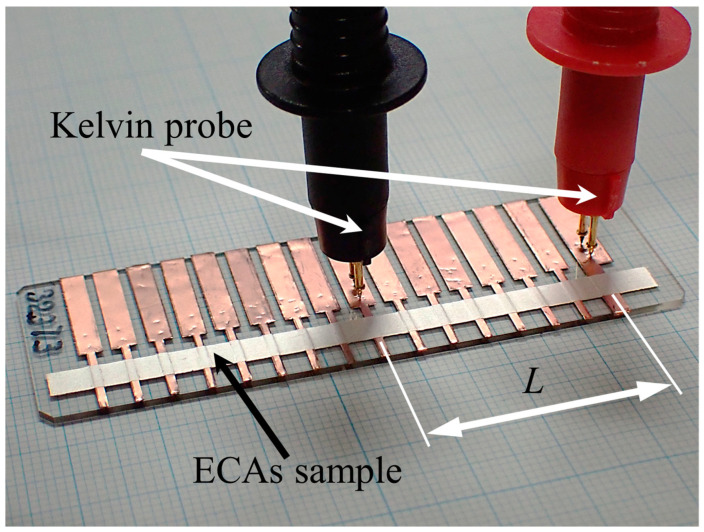
The electrical resistance measurement of the ECA sample printed on the substrate with Cu electrodes using a Kelvin probe.

**Figure A3:**
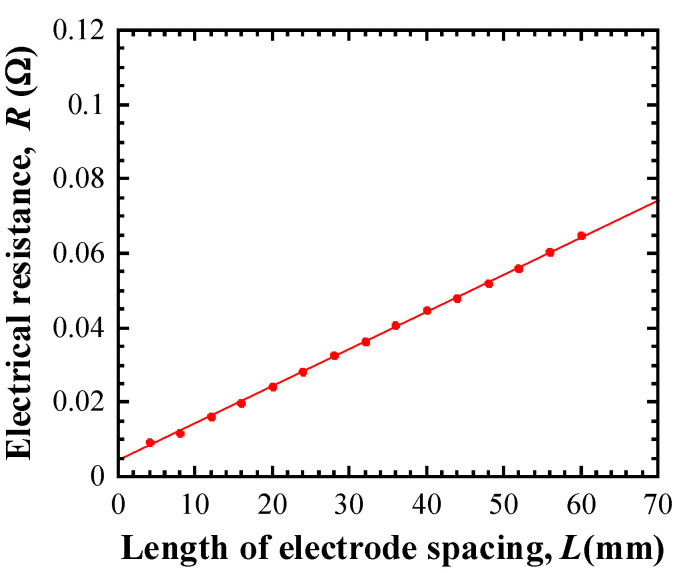
A result analyzing the relationship between the electrical resistance and electrode spacing.

## Data Availability

The original contributions presented in this study are included in the article. Further inquiries can be directed to the corresponding author.

## Appendix A

Before the preparation of substrates for measuring the interfacial electrical resistivity, the surfaces of the electrolytic and the rolled Cu foils, which were used as electrodes, were analyzed using XPS (AXIS-NOVA, Shimadzu, Kyoto, Japan).

Figure A1a shows the electrolytic Cu electrode analysis result of the survey scan. This analysis found no Cu on the surface of the electrode, but there was Zn. Analysis using a GD-OES spectrometer (GD-Profiler 2; HORIBA France SAS, Lyon, France) found the electrolytic Cu electrode had a surface layer of Zn about 15–20 nm thick. It was determined that the surface of the electrolytic Cu foil used in this study had been plated with Zn. Figure A1b shows the XPS spectrum for a rolled Cu electrode. It was confirmed that Cu was present on the electrode surface, indicating that no plating had been applied.

## Appendix B

The method for estimating the interfacial electrical resistivity between the ECAs and Cu electrodes is described in Section 2.5. A supplemental explanation of the experimental procedure is added here. The electrical resistance of the samples was measured using a Kelvin probe for the four-electrode measurement by changing electrode spacing, as shown in Figure A2. The measuring data can analyze the relationship between the electrical resistance and the electrode spacing by fitting Equation (1), indicated in Section 2.5, as shown in Figure A3. The interfacial electrical resistivity can be estimated using the intercept with the electrical resistance axis.
